# A Rare Cause of Hypoglycemia in Elderly Patients: Insulinoma

**DOI:** 10.7759/cureus.54002

**Published:** 2024-02-11

**Authors:** Ahmet Baris Dirim, Ahmet Seker

**Affiliations:** 1 Gastroenterological Surgery, University of Health Sciences, Adana City Training and Research Hospital, Adana, TUR

**Keywords:** whipple's triad, spleen-preserving distal pancreatectomy, kimura technique, insulinoma, hypoglycemia

## Abstract

Insulinoma is the most common pancreatic neuroendocrine tumor and is often solitary and benign. To make a diagnosis, high insulin levels must be demonstrated, and proinsulin and C-peptide measurements must be done. The presence of hypoglycemic measurements and symptoms in the 72-hour fasting test is diagnostic.

We present a case of a 91-year-old patient with no known diagnosis of diabetes mellitus who was admitted to the emergency department due to confusion. As a result of the clinical evaluation and differential diagnosis, it was determined that her complaint was due to hypoglycemia, and she was admitted to the internal medicine service for further examination, diagnosis, and treatment.

## Introduction

Insulinoma is a functional neoplasia originating from pancreatic beta cells and is the most common cause of organic hyperinsulinemic hypoglycemia in adults. Its incidence is one to four per million [1]. Of these tumors, 90% are benign, solitary, and intrapancreatic. Their dimensions are 80% below 2 cm [2]. About 30% of them are smaller than 1 cm and 10% are multiloculated [3].

The distinctive features of insulinomas were defined as the “Whipple triad” by Allen Whipple and Virginia Kneeland Frantz in 1930 [4]. Hypoglycemic symptoms, blood sugar levels lower than 55 mg/dl (3.05 mmol/L) during episodes, and regression of symptoms following glucose intake are the classic features of this triad. A total of 5-10% of insulinomas are malignant, and it is very difficult to control insulin secretion and hypoglycemia in these patients.

Clinical diagnosis of insulinoma is made by spontaneous measurement in patients with the Whipple triad or by documenting high serum insulin, C-peptide, and proinsulin levels during a 72-hour prolonged fasting test. As soon as a clinical diagnosis is made, the anatomical localization of the tumor should be made and it should be determined whether there is metastasis.

Treatment is mainly surgical. If the patient's clinical condition is suitable for surgery, surgical planning should be made once the diagnosis and tumor localization are confirmed. As in the case we present, the diagnosis of many patients may be delayed for months or even for years, and patients may receive psychiatric and neurological diagnoses considering that they are having a seizure [5].

## Case presentation

A 91-year-old female patient with no known diagnosis of diabetes mellitus, diagnosed with dementia and hypertension, was admitted to the emergency department due to confusion. In the evaluation, the blood sugar level of the patient, who had no acute neurological findings, was found to be 25 mg/dl, and her symptoms regressed with sugar intake. She was admitted to the internal medicine service for differential diagnosis and further examination. At the 5th hour of the 72-hour prolonged fasting test, serum glucose level was detected as 40 mg/dl (2.22 mmol/L) along with symptoms of hypoglycemia. Simultaneous serum insulin was 28.69 mU/L (reference range: 1.9-23) and the C peptide level was 4.25 µg/L (reference range: 0.9-7.1). Anterior pituitary hormone results and other biochemical values were normal. The patient, who was diagnosed with insulinoma, was started on a 10% dextrose infusion to prevent hypoglycemic attacks. Since diazoxide was not available in our country, verapamil 120 mg oral treatment was started.

**Figure 1:**
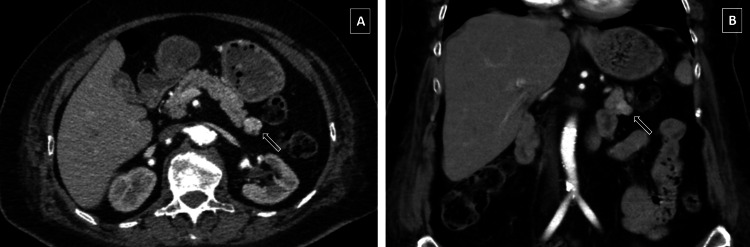
Contrast-enhanced CT scan of the abdomen. The white arrow points to the nodular lesion located in the tail of the pancreas. (A) Axial view. (B) Coronal view.

As a result of the radiological examinations, a dynamic pancreatic computed tomography (CT) scan revealed a "1.5 cm-sized nodular lesion with an exophytic extension in the tail of the pancreas, with slightly more contrast in the early arterial phase than in the pancreas in the coronal plane" (Figure 1). In the endoscopic ultrasonographic (EUS) examination, the pancreatic parenchyma and duct were observed to be normal.

**Figure 2:**
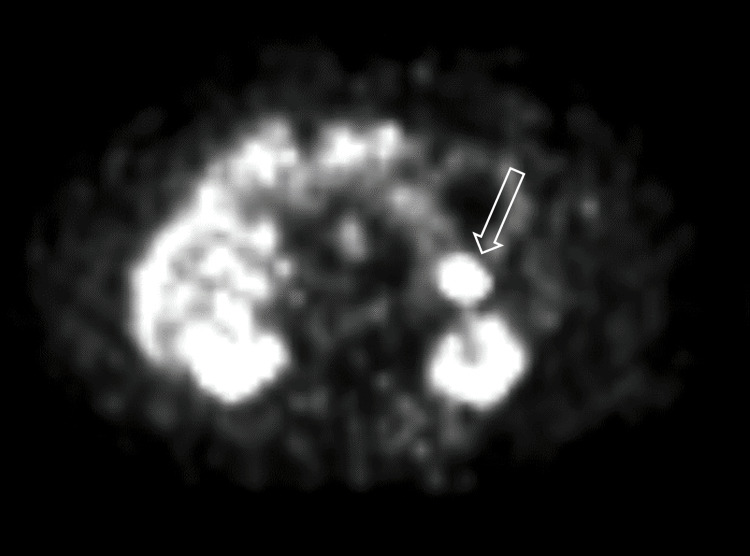
Gallium 68 uptake in the tail of the pancreas. The white arrow points to the nodular lesion (positron emission tomography view).

In positron emission tomography-CT (PET-CT), intense focal increased (maximum standard uptake value: 26) GA68-DOTA uptake was reported in a nodular lesion with a diameter of approximately 1.5 cm in the tail of the pancreas (Figure 2). The patient, who was dependent on dextrose infusion and medical treatment during his 40-day internal medicine hospitalization, was taken into surgical planning with the diagnosis of insulinoma.

**Figure 3:**
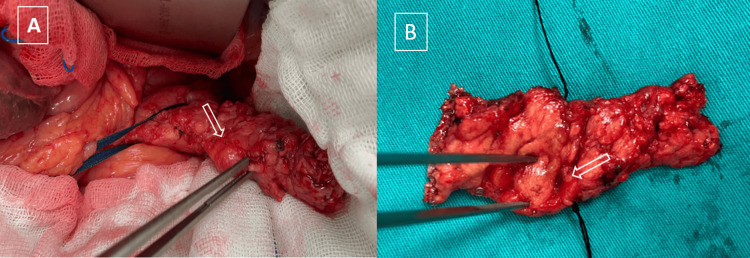
The white arrow points to the nodular lesion located in the tail of the pancreas. (A) Intraoperative view. (B) Surgical specimen.

By combining intraoperative ultrasonography (IOUS) and pancreatic palpation, a lesion located at the body-tail junction of the pancreas, as indicated by preoperative imaging, was detected. No signs of invasion were observed during the dissection of the peripancreatic surrounding tissues. Spleen-preserving distal pancreatectomy (Kimura technique) surgery was performed with no complications and a drain was placed (Figure 3). The insulin level measured intraoperatively after resection was found to be 6.56 mU/L within the normal reference range.

Intermittent insulin infusion was administered to the patient, who was hyperglycemic on postoperative (PO) day one. Pancreatic leakage did not develop in the following days. The patient, whose glucose measurements returned to normal levels, was discharged without any problems after the drain was removed on PO day seven. During outpatient follow-up, glucose levels were within normal limits and no complications were encountered.

## Discussion

Due to the symptoms being nonspecific and varying from patient to patient, the clinician's suspicion of the diagnosis of insulinoma and questioning the patient in this regard is the most important step for diagnosis. Since symptoms occur intermittently, tests performed during the asymptomatic period are not sufficient to diagnose or rule out insulinoma. Demonstration of a normal glucose level, when symptoms appear, will rule out the diagnosis of insulinoma. Clinical conditions such as malnutrition, adrenal insufficiency, and non-insulin-mediated hypoglycemia should be excluded before the fasting test [6]. Although the Whipple triad is the traditional diagnostic method, the main diagnosis is made with a 72-hour fasting test.

The next step after diagnosis is to determine the location of the tumor. The sensitivity of conventional examinations, i.e., ultrasound (US), CT, and magnetic resonance imaging (MRI), varies between 10% and 61%, 30% and 66%, and 37% and 71%, respectively [7]. When conventional examinations fail to localize the tumor, more expensive and more invasive methods (EUS, PET-CT, arteriography, transhepatic portal venous sampling-Imamura Doppman procedure) are often needed. Since localization studies in our patient's conventional imaging were not successful, the tumor site was localized with PET-CT and dynamic pancreas CT imaging. EUS, whose sensitivity is reported to be in the range of 70-95%, was reported normal in our patient [8].

Since insulinomas are rarely located outside the pancreas, some authors have suggested only excluding the presence of metastasis with preoperative conventional examinations and localizing the tumor during the operation. It is reported that when IOUS and palpation methods are used together, tumor detection sensitivity approaches 100%. However, there are more authors who believe that localizing the tumor before the operation directly affects the operation strategy, minimizes surgery-related trauma, and reduces unnecessary biopsy [9].

Surgical options can be classified as open or laparoscopic resection, distal pancreatectomy, or tumor enucleation. Since it is not possible to palpate the lesion in the laparoscopic surgical approach, it is necessary to ensure the localization of the lesion in preoperative preparation. At the same time, the sensitivity of laparoscopic IOUS is much lower than that performed in open surgery [10]. There are no specific morphological, genetic, or biochemical features that distinguish malignant insulinomas from benign ones.

Spleen-preserving distal pancreatectomy (Kimura procedure) surgery was performed with open surgery for the lesion, which was located at the body-tail junction of the pancreas and was not well-circumscribed. No complications were encountered in the postoperative follow-up, except for short-term hyperglycemia. Mehrabi et al. reported similar morbidity rates in open and closed surgery (35.4% and 32.8%, respectively), and found pancreatic fistula as the most common complication (open surgery (14.6%) vs. laparoscopic surgery (7.2%)) [11]. Pancreatic fistulas develop more frequently in enucleation surgery compared to the surgical method with resection, and since they have a low flow rate, they mostly regress with short-term conservative follow-up. Other common complications are abscess (3.4%) and pseudocyst (3%) for open and laparoscopic surgery [12].

## Conclusions

Insulinoma can be confused with many psychiatric and neurological diseases due to the neuroglycopenic symptoms that occur. In cases where conventional and advanced imaging methods are inadequate in tumor localization, invasive procedures and sometimes the location of the mass may need to be determined during the operation.
